# Human Cord Blood-Derived CD133^+^/C-Kit^+^/Lin^−^ Cells Have Bipotential Ability to Differentiate into Mesenchymal Stem Cells and Outgrowth Endothelial Cells

**DOI:** 10.1155/2016/7162160

**Published:** 2016-12-18

**Authors:** Carlos Cardenas, Ja-Young Kwon, Yong-Sun Maeng

**Affiliations:** ^1^Department of Obstetrics, Gynecology and Reproductive Sciences, Division of Reproductive Sciences, Yale School of Medicine, New Haven, CT, USA; ^2^Department of Obstetrics and Gynecology, Institute of Women's Life Medical Science, Yonsei University College of Medicine, Seoul, Republic of Korea

## Abstract

Recent evidence suggests that mononuclear cells (MNCs) derived from bone marrow and cord blood can differentiate into mesenchymal stem cells (MSCs) or outgrowth endothelial cells (OECs). However, controversy exists as to whether MNCs have the pluripotent capacity to differentiate into MSCs or OECs or are a mixture of cell lineage-determined progenitors of MSCs or OECs. Here, using CD133^+^/C-kit^+^/Lin^−^ mononuclear cells (CKL− cells) isolated from human umbilical cord blood using magnetic cell sorting, we characterized the potency of MNC differentiation. We first found that CKL− cells cultured with conditioned medium of OECs or MSCs differentiated into OECs or MSCs and this differentiation was also induced by cell-to-cell contact. When we cultured single CKL− cells on OEC- or MSC-conditioned medium, the cells differentiated morphologically and genetically into OEC- or MSC-like cells, respectively. Moreover, we confirmed that OECs or MSCs differentiated from CKL− cells had the ability to form capillary-like structures in Matrigel and differentiate into osteoblasts, chondrocytes, and adipocytes. Finally, using microarray analysis, we identified specific factors of OECs or MSCs that could potentially be involved in the differentiation fate of CKL− cells. Together, these results suggest that cord blood-derived CKL− cells possess at least bipotential differentiation capacity toward MSCs or OECs.

## 1. Introduction

Stem cells are a current focus of scientific research due to their plasticity and extensive self-renewal capacity and ability to differentiate into one or more committed descendants, including fully functional mature cells [1]. Stem cells can differentiate into many cell types, such as cardiomyocytes, vascular cells, neurons, and hepatocytes, both* in vitro* and* in vivo*. For this reason, regenerative medicine has become a potential therapy for degenerative diseases such as myocardial infarction, vascular diseases, motor neuron diseases, neurodegenerative diseases, and liver disease [2–7]. Classified according to their origin, stem cells may be embryonic stem cells, tissue-specific stem cells, mesenchymal stem cells (MSCs), or induced pluripotent stem cells. Because the use of embryonic stem cells is restricted due to ethical concerns, tissue-specific stem cells, MSCs, and induced pluripotent stem cells are the main cell types for tissue engineering.

Umbilical cord blood stem cells can differentiate into endothelial cells or MSCs* in vitro* and* in vivo* and improve poorly functioning organs [8–10]. Two types of endothelial cells cultured from human peripheral blood, endothelial progenitor cells and outgrowth endothelial cells (OECs), show comparable angiogenic capabilities [11]. OECs have outgrowth potential and may potentially be used for angiogenic therapies via transplantation with endothelial progenitor cells [12, 13]. Although MSCs are capable of differentiating into cells of different connective tissue lineages, such as bone, cartilage, and adipose tissue [10], the mechanisms underlying the differentiation of stem cells derived from human cord blood into MSCs or OECs are not fully understood.

To determine whether cord blood-derived mononuclear cells (MNCs) have the ability to differentiate into MSCs or OECs or are a mixture of cells containing cell lineage-determined progenitors of MSCs or OECs, we characterized the differentiation potency of CD133^+^/C-kit^+^Lin^−^ MNCs (CKL− cells) isolated from human umbilical cord blood using magnetic activated cell sorting. When CKL− cells were cultured on MSC- or OEC-conditioned medium, they preferred to differentiate into MSCs or OECs, respectively. Direct coculture of CKL− cells with OECs or MSCs also induced their differentiation into OECs or MSCs, which had the ability to form capillary-like structures in Matrigel or to differentiate into osteoblasts, chondrocytes, or adipocytes. Moreover, using microarray analysis, we identified the specific factors of OECs and MSCs that could direct the cell fate of CKL− cells.

## 2. Materials and Methods

### 2.1. Study Population and Sample Collection

Of the deliveries at our institute between June 2007 and March 2008, only those performed by cesarean section at 37–41 weeks of gestation were included in this study. Umbilical cord blood for CKL− cell isolation was obtained at the time of delivery after fetal expulsion. Pregnancies associated with premature rupture of membranes, fetal malformation, chromosome anomaly, multiple pregnancies, preeclampsia, hypertension, or renal or endocrine diseases were excluded from the study. The sampling and use of medical records for research purposes were performed with the consent of patients. This study was approved by the Yonsei University Hospital Review Board (4-2005-0186).

### 2.2. Isolation and Cultivation of CKL− Cells

We isolated endothelial progenitor cells from human umbilical cord blood. Blood samples (~50 mL each) were collected from fresh placentas with attached umbilical cords by gravity flow. MNCs were isolated by density gradient centrifugation over Biocoll (Biochrom, Berlin, Germany) for 30 min at 400 ×g and washed three times in phosphate buffered saline (PBS) (Biochrom). CKL− cells were purified by positive and negative selection with anti-CD133/C-kit/Lin^−^ microbeads (Miltenyi Biotec, Bergisch-Gladbach, Germany) using a magnetic cell sorter device (Miltenyi Biotec). Briefly, cord blood MNCs were incubated with anti-CD133 microbeads and unbounded antibodies were removed by cell washing. Cells incubated with anti-CD133 microbeads were processed for positive selection, according to the manufacturer's instructions. CD133+ fraction was then incubated with anti-C-kit microbeads and processed for running sensitive positive selection. For depletion of Lin+ cells from CD133+/C-kit+ fractions, cells were incubated with anti-Lin microbeads and applied on column. Unbound cells were washed out and collected. This fraction is CD133+/C-kit+/Lin−. Purity, as assessed by fluorescence activated cell sorting analysis, was >98%. CKL− cells were seeded onto 6-well plates coated with human fibronectin (Sigma, St. Louis, MO) in endothelial basal medium-2 (Clonetics, Cell Systems, St. Katharinen, Germany). The medium was supplemented with endothelial growth medium-2 (EGM-2; Clonetics, Cell Systems) containing fetal bovine serum, human VEGF-A, human fibroblast growth factor-B, human epidermal growth factor, IGF1, and ascorbic acid in appropriate amounts. CKL− cell identification was determined by staining cells with phycoerythrin- (PE-) conjugated human antibodies CD133-PE and C-kit-PE (BD Biosciences, Bedford, MA).

### 2.3. CKL− Cell Differentiation Assay

CKL− cells (5 × 10^5^ cells/well) were seeded on 6-well plates and cultured in OEC- or MSC-conditioned medium (100% EGM-2 (control), 12.5% conditioned medium + 87.5% EGM-2, 25% conditioned medium + 75% EGM-2, or 50% conditioned medium + 50% EGM-2). The medium was changed every 2 days. The day of differentiation was defined as the first day on which a differentiated colony was observed from the time of seeding. At least three assays were performed for each sample.

CKL− cells were cocultured with green fluorescent protein- (GFP−) transfected OECs or MSCs. After 3 days in culture, GFP^−^ OECs or MSCs began to appear among GFP^+^ OECs or MSCs. GFP^−^ OECs and MSCs differentiated from CKL− cells were confirmed by immunofluorescent staining for VE-cad antibody and alpha-smooth muscle actin (*α*-SMA), respectively.

After 5 days in culture, CKL− cells were harvested, diluted in cell culture medium to approximately 1 cell/100 *μ*L, and replated in 96-well cell culture plates. After 24 hours, the culture medium was changed to OEC- or MSC-conditioned medium (50%). On day 4, differentiated cells were defined and further expanded in 24-well, 60 mm plates until cell numbers were sufficient for immunostaining analyses. At least three assays were performed for each sample.

### 2.4. Semiquantitative RT-PCR Analysis

Total RNA was obtained from CKL− cells, OECs, and MSCs with TRIzol reagent. RNA samples (0.5–5 *μ*g) were used for RT-PCR. Briefly, target RNA was converted to cDNA by treatment with 200 units of reverse transcriptase and 500 ng oligo(dT) primer in 50 mM Tris-HCl (pH 8.3), 75 mM KCl, 3 mM MgCl_2_, 10 mM dithiothreitol, and 1 mM dNTPs for 1 h at 42°C. The reaction was quenched by heating for 15 min at 70°C. One *μ*L of the cDNA mixture was used for PCR amplification. PCR reactions contained 50 mM KCl, 10 mM Tris-HCl (pH 8.3), 1.5 mM MgCl_2_, 0.2 mM dNTPs, 2.5 units of* Taq* DNA polymerase, and 0.1 *μ*M of primers. Amplification was performed in a model PTC-200 thermal cycler under the following conditions: denaturation at 94°C for 5 min for the first cycle and for 30 s thereafter, annealing for 30 s at 55°C, and extension for 30 s at 72°C for 28 repetitive cycles. A final extension step proceeded for 10 min at 72°C. Each experiment was performed in quadruplicate. The primers used are described in Supplementary Table S1 in Supplementary Material available online at http://dx.doi.org/10.1155/2016/7162160.

### 2.5. Immunofluorescence Staining of CKL− Cells, OECs, and MSCs

Cells were fixed in 4% paraformaldehyde for 20 min, permeabilized in 0.1% Triton X-100/PBS, and then preincubated with blocking solution consisting of PBS containing 5% normal goat serum and 0.05% Tween-20. Cells were then incubated for 2 h in primary antibody [mouse anti-CD133, anti-C-kit, anti-vWF, anti-CDH5 (VE-cadherin), anti-*α*-SMA, or anti-PDGFR*β* (BD Pharmingen, San Diego, CA)]. Reactions were visualized by FITC/TRITC-conjugated anti-human secondary antibody (Vector Laboratories, Burlingame, CA). All samples were observed with a fluorescence microscope (Olympus, Tokyo, Japan).

### 2.6. Osteogenesis Assay

Confluent MSCs were cultured for 10 days in DMEM low-glucose medium with 10% fetal bovine serum, 1x GPS, and osteogenic supplements (1 *μ*M dexamethasone, 10 mM *β*-glycerophosphate, and 60 *μ*M ascorbic acid-2-phosphate). Differentiation into osteocytes was assessed by von Kossa staining.

### 2.7. Chondrogenesis Assay

Suspensions of MSCs were transferred into 15 mL polypropylene centrifuge tubes (500,000 cells/tube) and gently centrifuged. The resulting pellets were statically cultured in DMEM high-glucose medium with 1x GPS and chondrogenic supplements (1x insulin-transferrin-selenium, 1 *μ*M dexamethasone, 100 *μ*M ascorbic acid-2-phosphate, and 10 ng/mL TGF-*β*1). After 14 days, pellets were fixed in 4% buffered formalin overnight, embedded in paraffin, and sectioned (7 *μ*m). Differentiation into chondrocytes was assessed by safranin-O staining.

### 2.8. Adipogenesis Assay

Confluent MPCs were cultured for 10 days in DMEM low-glucose medium with 10% FBS, 1x GPS, and adipogenic supplements (5 *μ*g/mL insulin, 1 *μ*M dexamethasone, 0.5 mM isobutylmethylxanthine, and 60 *μ*M indomethacin). Differentiation into adipocytes was assessed by Oil Red O staining.

### 2.9. Oligonucleotide Microarrays

Total RNA (10 *μ*g) was hybridized to the HG-U133A 2.0 microarray (54675 human genes; Affymetrix, Santa Clara, CA) following the manufacturer's standard protocol for sample preparation and microarray processing. Expression data were analyzed using Microarray Suite version 5.0 (Affymetrix) and GenPlex v2.4 software (ISTECH Inc., Seoul, Republic of Korea).

### 2.10. Statistical Analyses

Data are shown as mean ± standard error (SE). Statistical comparisons between groups were performed using one-way analysis of variance followed by Tukey's post hoc tests.

## 3. Results

### 3.1. Differentiation of Human Cord Blood-Derived CKL− Cells into OECs or MSCs

**Figure 1:**
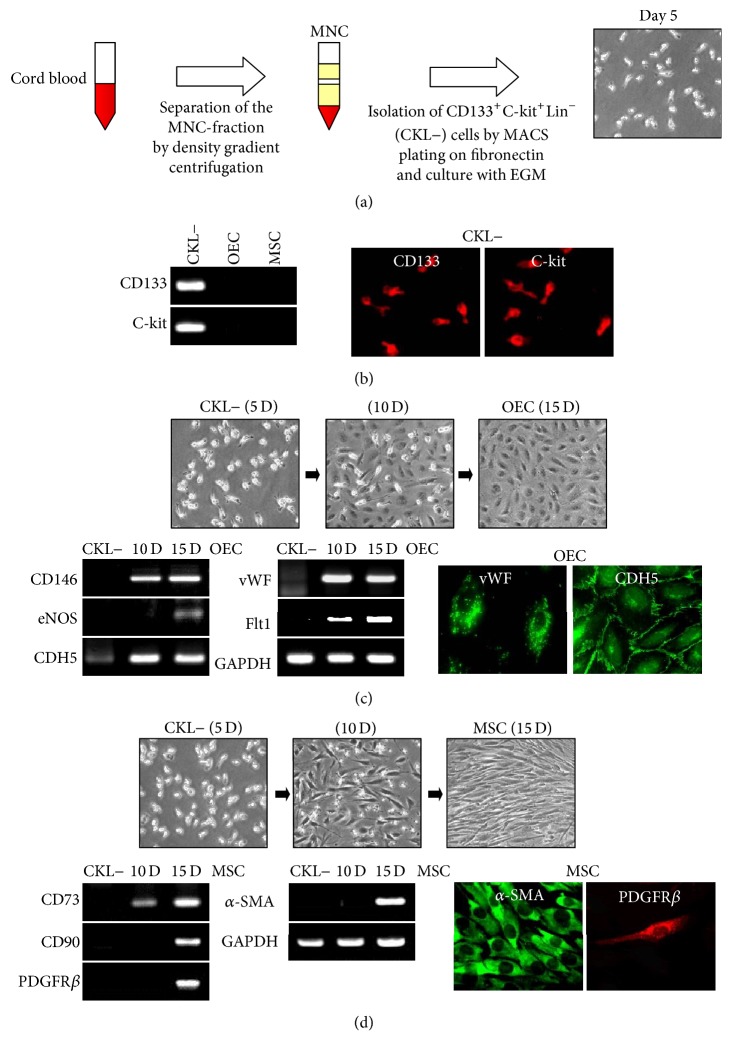
Isolation and characterization of CKL− cells from human umbilical cord blood. (a) CKL− cells were isolated by density gradient centrifugation over Biocoll for 30 min at 400 ×g, washed three times in PBS, and purified by positive selection with anti-CD133/C-kit/Lin− microbeads using a magnetic cell sorter device. (b) Total mRNA was isolated from CKL− cells, and gene expression was assessed by RT-PCR. CKL− cells were further characterized by immunofluorescent staining for CD133 and C-Kit. (c, d) CKL− cells differentiated into OECs and MSCs as confirmed by RT-PCR and immunofluorescent staining for OEC- and MSC-specific markers.

The MNC fraction was separated from cord blood using density gradient, and CKL− MNCs were sorted and purified (Figure 1(a)). The cellular phenotype of CKL− cells was confirmed by RT-PCR and immunostaining for CD133 and C-kit (Figure 1(b)). After 10 days in OECs or MSCs differentiation culture condition, CKL− cells spontaneously differentiated into OECs or MSCs as confirmed by cell morphology and expression of lineage-specific markers (Figures 1(c) and 1(d)).

### 3.2. CKL− Cells Have Multilineage Differentiation Potential Depending on Environmental Factors

**Figure 2:**
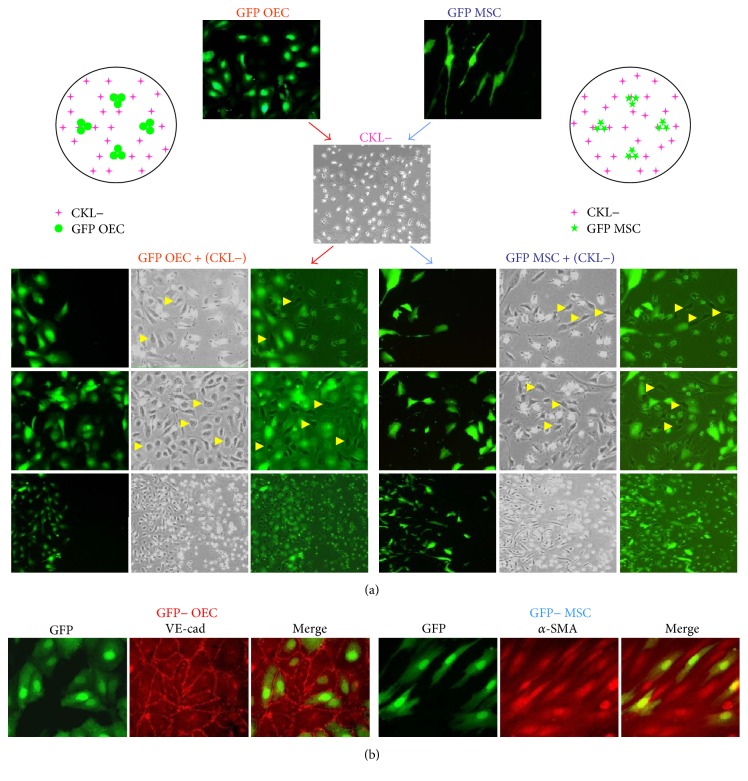
The microenvironment induces the differentiation of CKL− cells into OECs or MSCs. (a) CKL− cells were cocultured with GFP− transfected OECs or MSCs. After 3 days in culture, GFP^−^ OECs or MSCs began to appear among GFP^+^OECs or MSCs cells, respectively (arrowheads). (b) GFP^−^ OECs and MSCs differentiated from CKL− cells were confirmed by immunofluorescent staining for VE-cad and *α*-SMA antibody, respectively.

To investigate whether the environment regulates CKL− cell differentiation, CKL− cells were cocultured with GFP+ OEC or MSCs. Double labeling of OEC or MSC with GFP and CKL− cells with RFP is ideal experiment model; however, GFP or RFP-infection into CKL− cells induced significant detachment of CKL− cells from plate and we were unable to proceed with the experiment. Therefore, we only labeled the OEC or MSC with GFP− lentivirus and cocultured with nonlabeled CKL− cells. CKL− cells differentiated into the same cell lineage as the cells with which they were cocultured as confirmed by immunofluorescence staining for cell-specific markers (Figures 2(a) and 2(b)). These results indicate that the local environment determines the differentiation specification of CKL− cells.

**Figure 3:**
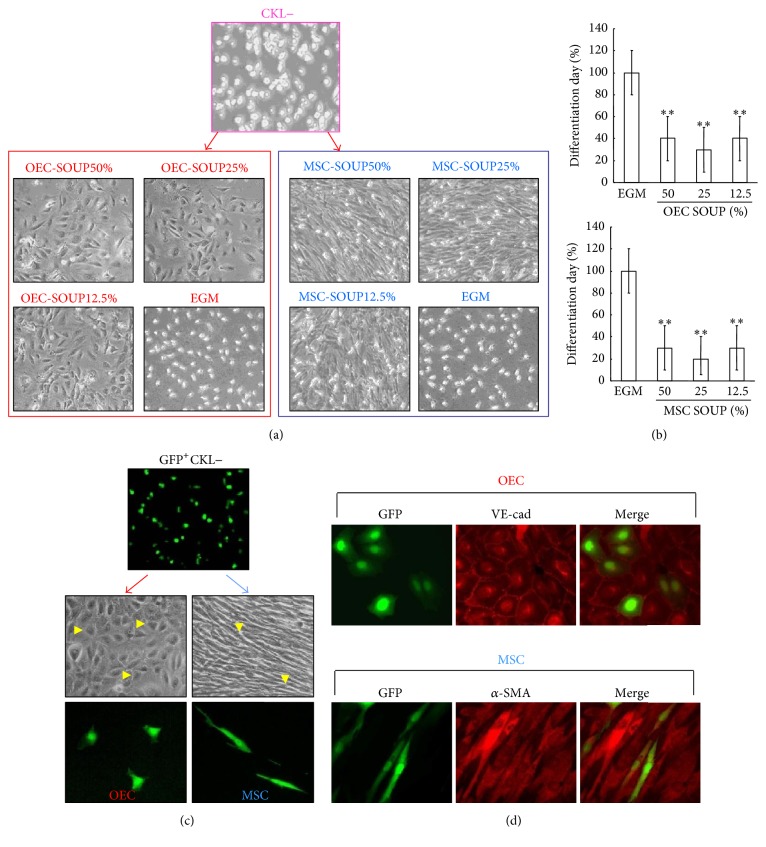
Soluble factors generated from OECs or MSCs accelerate CKL− cell differentiation. (a) CKL− cells were cultured in OEC- or MSC-conditioned medium in a dose-dependent manner (0, 12.5, 25, or 50%). (b) The day of differentiation, defined as the first day on which differentiated colonies were observed from the time of seeding, occurred significantly sooner in CKL− cells differentiated in conditioned medium compared with control conditions. ^*∗∗*^ *p* < 0.01 versus control. (c) GFP+ CKL− cells were cocultured with OECs or MSCs. Arrowheads indicate morphologically differentiated GFP^+^OECs or MSCs. (d) OECs or MSCs differentiated from GFP^+^CKL− cells were characterized by immunofluorescent staining using VE-cad or *α*-SMA antibody, respectively.

Next, CKL− cells were cultured on OEC- or MSC-conditioned medium in a dose-dependent manner to evaluate the effect of soluble factors on differentiation. The length of time to achieve differentiation was significantly reduced in CKL− cells cultured on conditioned medium compared with the control condition, suggesting that soluble factors produced by OECs or MSCs accelerate CKL− cell differentiation (Figures 3(a) and 3(b)). Moreover, GFP+ CKL− cells cocultured with OECs or MSCs differentiated into OECs or MSCs as confirmed by immunofluorescence staining for VE-cadherin or *α*-SMA, respectively (Figures 3(c) and 3(d)).

**Figure 4:**
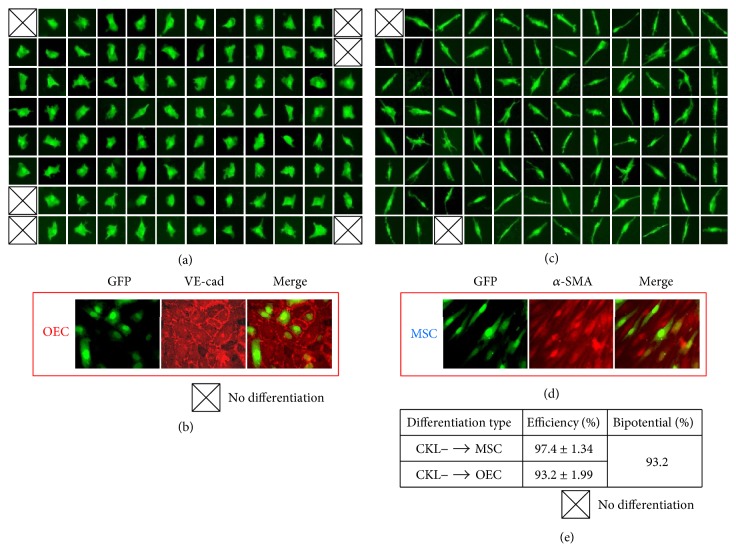
CKL− cells have multilineage differentiation potential depending on environmental factors. (a, c) Single GFP^+^ CKL− cells were on OEC- or MSC-conditioned medium. (e) The differentiation efficiency of CKL− cells into OECs and MSCs on day 4 was 93.22% and 97.37%, respectively. (b, d) The phenotype of differentiated GFP^+^OECs or MSCs was confirmed by immunofluorescent staining for VE-cadherin or *α*-SMA antibody, respectively.

CKL− cells did not proliferate in* in vitro* culture conditions. Therefore, to further determine whether CKL− cells have the potential to differentiate into multiple lineages depending on environmental cues or are a mixture of cells with monolineage differentiation potential toward OECs or MSCs, single GFP+ CKL− cells were cultured on OEC- or MSC-conditioned medium (Figures 4(a) and 4(c)). On day 4, 93.2% of cells cultured on OEC-conditioned medium and 97.4% of cells cultured on MSC-conditioned medium exhibited OEC and MSC phenotypes, respectively (Figure 4(e)). The phenotype of differentiated GFP+ OECs or MSCs was confirmed by immunofluorescence staining for VE-cadherin or *α*-SMA, respectively (Figures 4(b) and 4(d)). It is well known that lentivirus integrates into host cell genome and is maintained for a long time. Therefore many researchers use GFP− lentivirus for cell tracing. However, cells can lose lentivirus GFP− fluorescence during over multiple passages. In our experiment, differentiated GFP+ single cells were expanded until cell numbers were sufficient for immunostaining, during which some cells lost GFP. Therefore, some GFP negative cells are present among the GFP positive cells (Figures 4(b) and 4(d)).

Based on these results, maximum 93.2% (=93.2%∩97.4%) of CKL− cells have bilineage differentiation potential. As a result, CKL− cells had the potential to differentiate into both OECs and MSCs (Figure 4(e)). Collectively, these results suggest that CKL− cells have multilineage differentiation potential depending on environmental factors.

### 3.3. Functional Characterization of OECs and MSCs Differentiated from CKL− Cells

**Figure 5:**
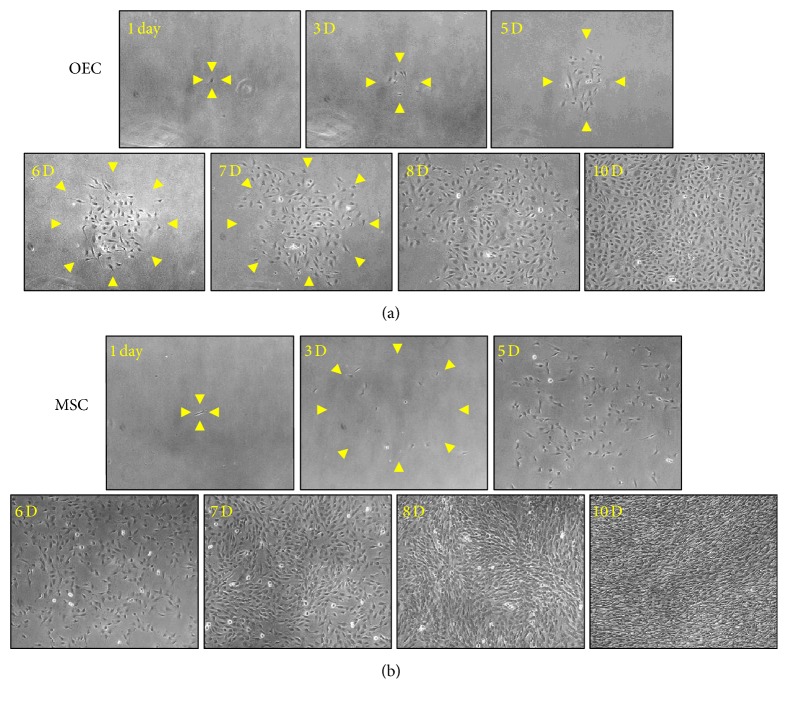
Representative colony formation assay of OECs or MSCs derived from CKL− cells. (a, b) Single OECs or MSCs differentiated from CKL− cells were cultured, and cell growth was analyzed at each time point. Arrowheads indicate the boundary of colonies.

Progenitor cells are defined and distinguished by their clonogenic and proliferative potential. We analyzed the proliferative kinetics of OECs and MSCs derived from CKL− cells. We initially plated single OECs or MSCs differentiated from CKL− cells to test whether they would form a colony and grow to confluence. Interestingly, the cell progeny of single OECs or MSCs formed colonies of various sizes before growing to confluence (Figures 5(a) and 5(b)).

**Figure 6:**
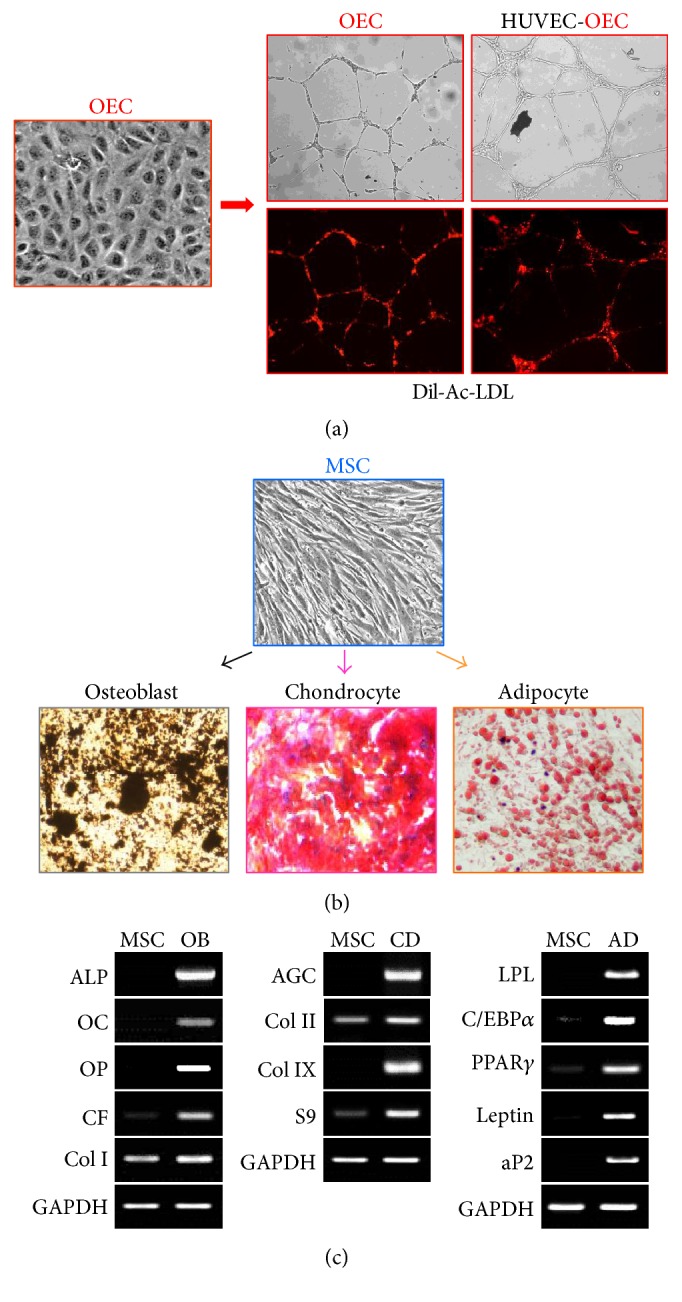
Functional characterization of OECs and MSCs. (a) Imaging of Dil-Ac-LDL-labeled OECs cultured on Matrigel-coated wells demonstrating capillary-like structure formation. (b) After incubation for 2-3 weeks in each differentiation medium, MSCs stained positively for calcium deposition (von Kossa staining), chondrocyte matrix (safranin-O staining), and lipid vacuoles (Oil Red O staining), indicating osteogenic, chondrogenic, and adipogenic differentiation, respectively. (c) Total mRNA was isolated from osteoblasts, chondrocytes, and adipocytes, and specific marker gene expression profiles were assessed by RT-PCR. OB: osteoblast, CD: chondrocyte, and AD: adipocyte.

We next tested whether the OECs derived from CKL− cells could incorporate Dil-acetylated low-density lipoprotein (Dil-Ac-LDL) and form capillary-like structures in Matrigel, which is characteristic of endothelial cells. Images of OECs differentiated from adherent CKL− cells show the uniform incorporation of Dil-Ac-LDL and the formation of capillary-like structures in Matrigel, similar to blood vessel endothelial cells (Figure 6(a)).

Next, the osteogenic potential of MSCs derived from CKL− cells was assessed by culturing cells under optimal conditions for inducing osteogenic differentiation. When differentiated under osteogenic conditions, the spindle shape of MSCs derived from CKL− cells flattened, broadened, and formed a mineralized matrix as shown by von Kossa staining (Figure 6(b), left panel). An osteoblastic phenotype was also evidenced by the expression of marker genes alkaline phosphatase (ALP), osteocalcin (OC), osteopontin (OP), runt-related transcription factor 2 (CBFA1:CF), and type I collagen (Col I) (Figure 6(c), left panel).

Furthermore, the chondrogenic potential of MSCs derived from CKL− cells was examined by the pelleted micromass system in serum-free chondrogenic medium. After 2 weeks of differentiation, the accumulation of sulfated proteoglycans was visualized by safranin-O staining (Figure 6(b), middle panel). Expression of mRNA for aggrecan (AGC), type II collagen (Col II), type IX collagen (Col IX), and Sox9 (S9), which are marker genes for chondrocytes, was detected by RT-PCR after 14 days of induction (Figure 6(c), middle panel).

Finally, CKL− cells were cultured in adipogenic medium to assess the potential of MSCs to become adipocytes. Morphologic changes and the formation of neutral lipid vacuoles were visualized by staining with Oil Red O (Figure 6(b), right panel). An adipogenic phenotype was also detected by the expression of marker genes lipoprotein lipase (LPL), CCAAT/enhancer-binding protein alpha (C/EBP*α*), peroxisome proliferator-activated receptor gamma (PPAR*γ*), leptin, and adipocyte P2 (aP2) (Figure 6(c), right panel).

### 3.4. Cell-Specific Molecules Regulate CKL− Cell Differentiation toward OECs or MSCs

**Figure 7:**
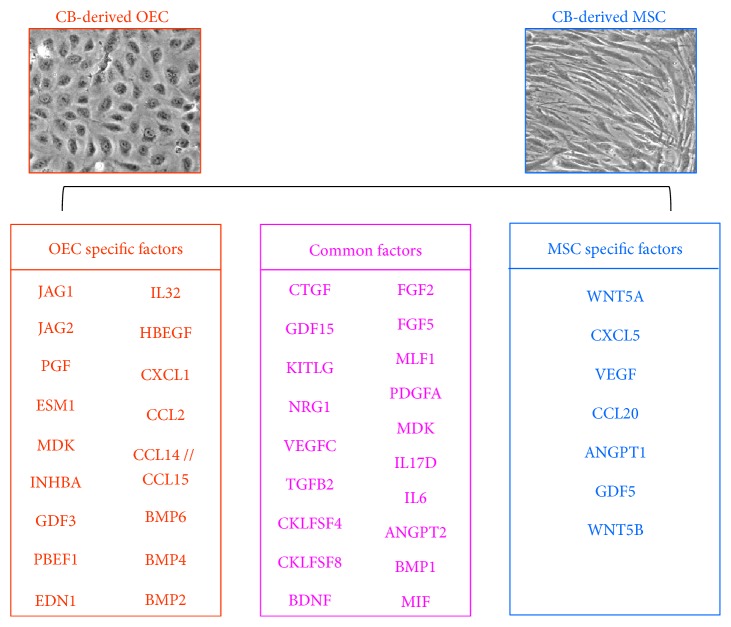
CKL− cell fate may be determined by specific molecules. Gene expression profiles were analyzed and compared between OECs and MSCs, and cell-specific upregulated genes are shown.

As CKL− cell differentiation fate was determined by the conditioned medium in which cells were cultured, we hypothesized that paracrine/endocrine factors provided by OECs and MSCs are involved in driving the differentiation of CKL− cells into specific lineages. To address this, we performed comparative analysis of gene expression between OECs and MSCs to identify cell-specific expression of genes encoding soluble factors. Genes expressed more highly in OECs were Jagged 1 (JAG1), JAG2, placental growth factor (PGF), endothelial cell-specific molecule 1 (ESM1), neurite growth-promoting factor 2 (MDK), inhibin beta A (INHBA), growth differentiation factor-3 (GDF3), pre-B-cell colony-enhancing factor 1 (PBEF1), endothelin 1 (EDN1), interleukin 32 (IL32), heparin-binding EGF-like growth factor (HBEGF), chemokine (C-X-C motif) ligand 1 (CXCL1), chemokine (C-C motif) ligand 2 (CCL2), CCL14, CCL15, bone morphogenetic protein 2 (BMP2), BMP4, and BMP6, whereas genes expressed more highly in MSCs were wingless-type MMTV integration site family, member 5A (WNT5A), CXCL5, vascular endothelial growth factor (VEGF), CCL20, angiopoietin 1 (ANGPT1), growth differentiation factor 5 (GDF5), and WNT5B (Figure 7). Thus, these cell-specific factors secreted in the microenvironment may direct CKL− cell differentiation toward specific lineages.

## 4. Discussion

The primary source of stem or progenitor cells for research purposes has shifted from bone marrow to umbilical cord blood, which is easily accessible, does not involve invasive procedures for collection, can be stored for longer durations, and is immune-tolerant. Although once a discarded material, umbilical cord blood is now considered an alternative source for stem/progenitor cell transplantation and therapy due to its hematopoietic and mesenchymal components [14–17]. However, the yield of progenitor cells of interest from cord blood samples is variable and often unsatisfactory, limiting their therapeutic use. To overcome this hurdle, a better understanding of the primitive cell population in umbilical cord blood is imperative for optimizing cell expansion. Furthermore, although the differentiation capacity of cord blood MNCs has been extensively studied [18–20], little is known about whether MNCs have the pluripotent capacity to differentiate into MSCs or OECs or are a mixture of progenitor cells with determined MSC or OEC fates.

Previous studies report that adult peripheral blood and cord blood CKL− MNCs can give rise to MSCs and OECs [8, 21–26]. Consistently, we found that CLK- MNCs purified from cord blood were able to differentiate into both MSCs and OECs with demonstrable biological* in vitro* and* in vivo* function. The differentiation fate of CKL− MNCs largely depended on the type of cells with which MNCs were cocultured or from which conditioned medium was collected. These findings suggest that environmental factors, such as cell-to-cell contact or soluble factors secreted from surrounding cells, direct the differentiation of CKL− cells. Furthermore, to determine whether CKL− MNCs are a mixture of MSC and OEC progenitors or have multipotent differentiation capacity toward OECs or MSCs depending on the* in vivo* milieu, we cultured single CKL− cells in either MSC- or OEC-derived conditioned medium. We found that CKL− MNCs have bipotential ability to generate MSCs and OECs, most likely due to the influence of environmental factors.

Various cues within the microenvironment such as cell-cell contact, mechanical forces, soluble factors, and extracellular interactions are involved in the fate-determination steps of stem cells [27–30]. To date, however, soluble factors that contribute to lineage-specific differentiation of CKL− cells are not well studied. As we found that CKL− MNC differentiation depended on environmental factors such as the presence of specific cell types or conditioned medium, we hypothesized that soluble factor genes highly expressed in MSCs or OECs could determine CKL− cell differentiation fate. Based on gene expression profiling, we found that MSCs showed high gene expression of WNT5A, CXCL5, VEGF, CCL20, ANGPT1, GDF5, and WNT5B, whereas OECs showed high gene expression of JAG1, JAG2, PGF, ESM1, MDK, INHBA, GDF3, PBEF1, EDN1, IL32, HBEGF, CXCL1, CCL2, CCL14, CCL15, BMP2, BMP4, and BMP6. Likewise, others have reported the upregulation of PGF [31], ESM1 [32], GDF3 [33], CXCL1 [34], BMP2, and BMP4 [35] in cord blood OECs. Previous studies also show that MSCs secrete VEGF to promote differentiation of neuronal precursors [36, 37] and CCL20 to modulate immune response [38, 39]. JAG1 and JAG2, which are NOTCH ligands, are known to play a pivotal role in the angiogenesis of various organs and injured arteries [40–44]. Furthermore, we recently showed that the expression of endothelial genes Delta-like 1 (DLL1) and JAG1 is closely related to the differentiation and angiogenic function of CKL− cells [8]. Nonetheless, while these specific genes for secreted factors identified may direct differentiation, it is also likely that these gene expressions are the result and not the cause of the differentiation. Therefore, further investigation is needed to confirm their role and mechanisms by which these molecules regulate lineage-specific differentiation.

In conclusion, CKL− MNCs in umbilical cord blood possess the ability to differentiate into MSC and OEC lineages depending on specific microenvironmental factors. This result suggests that CKL− MNCs are excellent sources of EC and MSC for the tissue regeneration.

## 5. Conclusions

This study demonstrates that human umbilical cord blood-derived CKL− cells can differentiate into OECs or MSCs and have multilineage differentiation potential. The microenvironment induces the differentiation of CKL− cells into OECs or MSCs and soluble factors generated from OECs or MSCs accelerate CKL− cell differentiation. Our data reveals the tremendous potential of CKL− MNCs as excellent sources of EC and MSC for the tissue regeneration.

## Supplementary Material

Genes and PCR primer sequence.
