# Supplementary material for: Human Cord Blood-Derived CD133+/C-Kit+/Lin− Cells Have Bipotential Ability to Differentiate into Mesenchymal Stem Cells and Outgrowth Endothelial Cells
Source: Stem Cells Int. 2016 Dec 18;2016:7162160. doi: 10.1155/2016/7162160 (PMC5203918; doi:10.1155/2016/7162160)
Supplement: Supplementary file 1 — Genes and PCR primer sequence. [file 7162160.f1.docx]

Supplementary Table S1

| eNOS | Sense- tggtcaactatttcctgtcc | OP | Sense- ctaggcatcacctgtgccatacc |
| --- | --- | --- | --- |
|  | Antisense- accacgtcatactcatccat |  | Antisense- cagtgaccagttcatcagattcatc |
| CD133 | Sense- cccgcaggagtgaatctttt | CF | Sense- cagtagatggacctcgggaa |
|  | Antisense-aggaaggactcgttgctggt |  | Antisense- gaggcagaagtcagaggtgg |
| c-kit | Sense- tctctttaggaagcagcccc | Col I | Sense- ccccctccccagccacaaaga |
|  | Antisense- acatttcagcaggtgcgtgt |  | Antisense- tcttggtcggtggtggactct |
| vWF | Sense- cccctgggttacaaggaaga | AGC | Sense- tcaggagggctggaacaagtacc |
|  | Antisense- gcgtctcatcacgcttcagt |  | Antisense- ggaggtggtaattgcagggaaca |
| CD146 | Sense- gtggtcatcgtggctgtgat | Col II | Sense- tttcccaggtcaagatggtc |
|  | Antisense- gatctcctgcttccctgagc |  | Antisense- cttcagcacctgtctcacca |
| Flt1 | Sense- ggtcttacggagtattgctg | Col IX | Sense- ccccctccccagccacaaaga |
|  | Antisense- ctttcttttgggtctctgtg |  | Antisense- tcttggtcggtggtggactct |
| CD73 | Sense- gagtgggtggtcagaaaata | S9 | Sense- ggttgttggagctttcctca |
|  | Antisense- tgcacactgtttttaaggtg |  | Antisense- tagcctccctcactccaaga |
| CD90 | Sense- taacagtcttgcaggtctcc | LPL | Sense- tcaatcacagcagcaaaacc |
|  | Antisense- aaggcggataagtagaggac |  | Antisense- ccacatctccaagtcctctc |
| α-SMA | Sense- gccgagatctcactgactac | C/EBPα | Sense- ctggagctgaccagtgacaa |
|  | Antisense- ggtccttcctgatgtcaata |  | Antisense- ccaagaattctcccctcctc |
| PDGFRβ | Sense- ctgcttctcgagagactgtt | GAPDH | Sense- atggggaaggtgaaggtcg |
|  | Antisense- gtgttgacttcattcagggt |  | Antisense-ggggtcattgatggcaacaata |
| CDH5 | Sense- tcaccctgctcatcttcctg | PPARγ | Sense- ttcagaaatgccttgcagtg |
|  | Antisense- catcgtagctggtggtgtcc |  | Antisense- gggctccataaagtcaccaa |
| ALP | Sense- tggagcttcagaagctcaacacca | leptin | Sense- acagaaagtcaccggtttgg |
|  | Antisense- atctcgttgtctgagtaccagtcc |  | Antisense- tggcttagaggagtcaggga |
| OC | Sense- catgagaagccctcaca | aP2 | Sense- tactgggccaggaatttgac |
|  | Antisense- agagcgacaccctagac |  | Antisense- tcaatgcgaacttcagtcca |
